# *APOE* ε4 carriers share immune-related proteomic changes across neurodegenerative diseases

**DOI:** 10.1038/s41591-025-03835-z

**Published:** 2025-07-15

**Authors:** Artur Shvetcov, Erik C. B. Johnson, Laura M. Winchester, Keenan A. Walker, Heather M. Wilkins, Terri G. Thompson, Jeffrey D. Rothstein, Varsha Krish, Farhad B. Imam, The Global Neurodegeneration Proteomics Consortium (GNPC), Artur Shvetcov, Erik C. B. Johnson, Laura M. Winchester, Keenan A. Walker, Terri G. Thompson, Jeffrey D. Rothstein, Varsha Krish, Farhad B. Imam, Jeffrey M. Burns, Chad Slawson, Caitlin A. Finney, Jeffrey M. Burns, Russell H. Swerdlow, Chad Slawson, Caitlin A. Finney

**Affiliations:** 1 Neurodegeneration and Disease Modelling Research Group, Westmead Institute for Medical Research, The University of Sydney, Westmead, New South Wales Australia; 2 School of Medical Sciences, Faculty of Medicine and Health, The University of Sydney, Sydney, New South Wales Australia; 3 Department of Neurology, Emory University, Atlanta, GA USA; 4 Goizueta Alzheimer’s Disease Research Centre, Emory University, Atlanta, GA USA; 5 Department of Psychiatry, University of Oxford, Oxford, UK; 6 Laboratory of Behavioral Neuroscience, National Institute on Aging, Intramural Research Program, Baltimore, MD USA; 7 University of Kansas Alzheimer’s Disease Research Centre, University of Kansas Medical Center, Kansas City, KS USA; 8 Department of Neurology, University of Kansas School of Medicine, Kansas City, KS USA; 9 Department of Biochemistry and Molecular Biology, University of Kansas Medical Center, Kansas City, KS USA; 10 OnPoint Scientific, Inc., San Diego, CA USA; 11 Brain Science Institute, Johns Hopkins University School of Medicine, Baltimore, MD USA; 12 Department of Neurology, Johns Hopkins University School of Medicine, Baltimore, MD USA; 13 Gates Ventures, Seattle, WA USA; 14 Department of Cell Biology and Physiology, University of Kansas Medical Center, Kansas City, KS USA; 15 University of Kansas Cancer Center, University of Kansas Medical Center, Kansas City, KS USA

**Keywords:** Neurodegeneration, Disease genetics

## Abstract

The *APOE* ε4 genetic variant is the strongest genetic risk factor for late-onset Alzheimer’s disease (AD) and is increasingly being implicated in other neurodegenerative diseases. Using the Global Neurodegeneration Proteomics Consortium SomaScan dataset covering 1,346 cerebrospinal fluid (CSF) and 9,924 plasma samples, we used machine learning-based proteome profiling to identify an *APOE* ε4 proteomic signature shared across individuals with AD, frontotemporal dementia (FTD), Parkinson’s disease dementia (PDD), Parkinson’s disease (PD), amyotrophic lateral sclerosis (ALS) and nonimpaired controls. This signature was enriched in pro-inflammatory immune and infection pathways as well as immune cells, including monocytes, T cells and natural killer cells. Analysis of the dorsolateral prefrontal cortex proteome for 262 donors from the Accelerating Medicines Partnership for AD UPenn Proteomics Study revealed a consistent *APOE* ε4 phenotype, independent of neurodegenerative pathology, including amyloid-β tau and gliosis for all diseases, as well as TDP-43 in ALS and FTD cases, and α-synuclein in PD and PDD cases. While systemic proteomic changes were consistent across *APOE* ε4 carriers, their relationship with clinical and lifestyle factors, such as hypertension and smoking, varied by disease. These findings suggest *APOE* ε4 confers a systemic biological vulnerability that is necessary but not sufficient for neurodegeneration, emphasizing the need to consider gene–environment interactions. Overall, our study reveals a conserved *APOE* ε4-associated pro-inflammatory immune signature persistent across the brain, CSF and plasma irrespective of neurodegenerative disease, highlighting a fundamental, disease-independent biological vulnerability to neurodegeneration. This work reframes *APOE* ε4 as a pleiotropic immune modulator rather than an AD-specific risk gene, providing a foundation for precision biomarker development and early intervention strategies across neurodegenerative diseases.

## Abstract (web summary)

Over 11,000 plasma and cerebrospinal fluid proteomic samples reveal that the APOE ε4 genetic variant is associated with a systemic pro-inflammatory signature in a variety of neurodegenerative diseases.

## Main

The ε4 variant of the apolipoprotein E (*APOE* ε4) gene is well-established as the largest genetic risk factor of late-onset Alzheimer’s disease (AD)^1^. Growing evidence, however, indicates that *APOE* ε4 carriage may also have a role in other age-associated neurodegenerative diseases. Studies have linked *APOE* ε4 to increased risk and lower age of onset of frontotemporal dementia (FTD)^2–6^, Parkinson’s disease (PD)^5,7,8^ and amyotrophic lateral sclerosis (ALS)^9,10^. *APOE* ε4 is also linked to a faster rate of cognitive decline and poor cognition in PD, increasing the risk of PD dementia (PDD)^11–16^. Despite the deleterious impact of *APOE* ε4, little is known about the biological mechanisms underlying this effect and if, or how, it changes across the different neurodegenerative diseases. We recently showed that *APOE* ε4 carriers, irrespective of cognitive status in AD and mild cognitive impairment, had the same proteomic signature in the cerebrospinal fluid (CSF) associated with a pro-inflammatory immune molecular phenotype^17^. However, whether this extends to other neurodegenerative diseases is unknown.

**Fig. 1:**
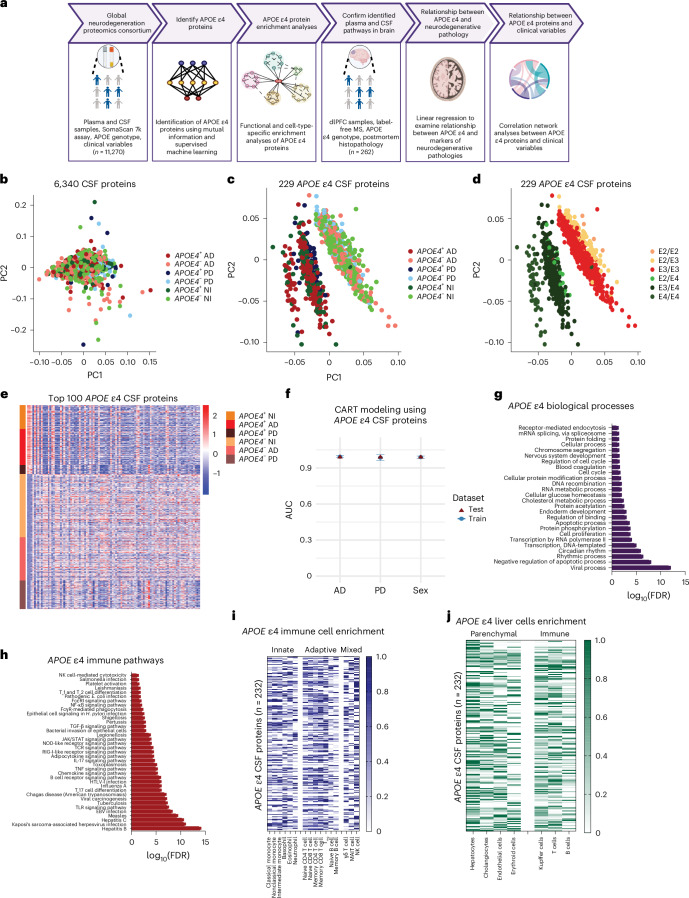
Study design and characterization of the CSF proteome signature in *APOE* ε4 carriers. **a**, Study design using the GNPC and AMP-AD UPenn Proteomics Study cohorts for identifying and characterizing systemic proteome changes in *APOE* ε4 carriers. Panel **a** is created with BioRender.com. **b**, PCA of all 6,340 measured CSF proteins showing no clear clustering. **c**, PCA of 229 *APOE* ε4 CSF proteins identified using mutual information that shows clustering based on the presence or absence of *APOE* ε4 allele rather than specific neurodegenerative disease. **d**, PCA of 229 *APOE* ε4 CSF proteins showing that clustering is based on the specific *APOE* genotype and number of *APOE* ε4 alleles (Supplementary Table 1 lists the distribution of *APOE* ε4 cases). **e**, Heatmap visualizing the upregulation (red) and downregulation (blue) of proteins within the *APOE* ε4 CSF proteome signature of 229 proteins, which shows distinctions based on the presence or absence of an *APOE* ε4 allele rather than disease. **f**, Supervised machine learning modeling using CART showing mean AUC ± s.d. across fivefold repeated five times. Models were trained and validated on a 70% training dataset and tested using a 30% withheld testing dataset. **g**, Functional enrichment analysis of PANTHER biological processes enriched for *APOE* ε44 CSF proteins showing the most significant (FDR = 9.34 × 10^−13^) enrichment for viral processes. **h**, Given the most significant enriched biological process was viral processes, we performed a functional enrichment analysis of KEGG immune-related pathways enriched for *APOE* ε4 CSF proteins, showing significant (FDR < 0.05) enrichment for immune, infection and pro-inflammatory pathways. **i**, Immune cell-type-specific enrichment analysis of *APOE* ε4 CSF proteins showing involvement across the innate, adaptive and innate-like T cells and lymphoid cells (mixed). **j**, Liver cell-type-specific enrichment analysis of *APOE* ε4 CSF proteins showing involvement across parenchymal and immune cells. Cell-type-specific enrichments are based on single-cell RNA-sequencing data from the Human Protein Atlas^19^. Plot shows min–max scaling of protein-coding transcripts per million for each identified protein in the *APOE* ε4 CSF signature. AUC, area under the curve; NI, nonimpaired controls; FcγR, Fc gamma R; FcεRI, Fc epsilon RI; NOD, nucleotide oligomerization domain; RIG, retinoic acid-inducible gene; HTLV-1, human T cell leukemia virus type 1.

To identify systemic proteomic changes associated with *APOE* ε4 carriers who develop neurodegenerative diseases, we used the Global Neurodegeneration Proteomics Consortium (GNPC) dataset. Here the plasma and CSF proteome were assessed using the SomaScan assay for 11,270 *APOE* ε4 carriers and noncarriers with AD, FTD, PD, PDD, ALS and nonimpaired controls. Using supervised machine learning, we identified and characterized an *APOE* ε4 proteome signature across the CSF and plasma. We then confirmed whether the same *APOE* ε4-enriched pathways in the periphery were mirrored in the brains of carriers and noncarriers. The dorsolateral prefrontal cortex (dlPFC) proteome of 262 AD, FTD, PDD, PD, ALS and nonimpaired control donors from the Accelerating Medicines Partnership for AD (AMP-AD) UPenn Proteomics Study was measured using label-free mass spectrometry (MS). In these samples, we also examined postmortem histopathological markers, including the presence of amyloid-β plaques, tau neurofibrillary tangles, gliosis and angiopathy. In FTD and ALS cases, we also examined TDP-43, while in PD and PDD, we examined α-synuclein. Lastly, to assess potential interactions between proteins in the *APOE* ε4 signature and environmental variables across the diseases, we performed a correlation network analysis between proteins and 18 clinical and lifestyle variables collected in the GNPC dataset, such as hypertension, smoking, and diabetes (Supplementary Table 1 and Fig. 1a).

## Results

CSF proteome profiling reveals a distinct signature in *APOE* ε4 carriers

**Extended Data Fig. 1:**
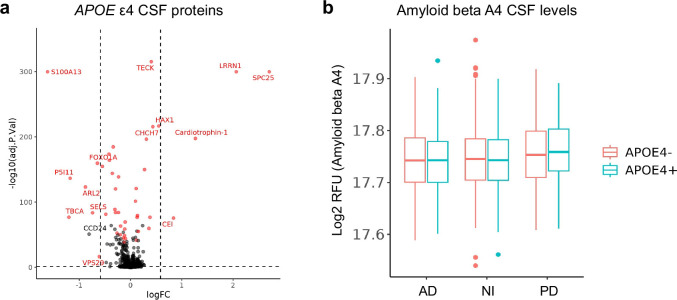
Further characterization of the *APOE* ε4 CSF protein signature. (**a**) Volcano plots showing fold change and adjusted p values for the *APOE* ε4 CSF proteins. Red dots indicate the proteins with the highest mutual information value (>0.1) for reference to the feature selection method used to identify *APOE* ε4 proteins. Differential protein abundance was assessed using linear modeling and empirical Bayes moderation. All tests were two-sided, and p-values were corrected for multiple comparisons using the Benjamini-Hochberg FDR method. Volcano plots display log_2_ fold change on the x-axis and -log₁₀-transformed adjusted p-values on the y-axis, with thresholds set at log_2_(FC) = ± 0.585 and FDR-adjusted p < 0.05. (**b**) Box plots showing amyloid beta A4 protein CSF levels in relative fluorescent units (RFU) across *APOE* ε4 carriers and non-carriers with AD, PD, and non-impaired controls. The center line represents the median, the bounds of the box indicate the 25th and 75th percentiles (interquartile range; IQR), and the whiskers extend to the minimum and maximum values within 1.5× IQR. Data points outside this range are plotted individually as outliers. N = 526 AD, 247 PD, and 573 non-impaired control individuals. Abbreviations: AD: Alzheimer’s disease; NI: non-impaired controls; PD: Parkinson’s disease.

We used SomaScan (6,340 proteins measured per sample) proteomic data from the GNPC dataset to profile the CSF proteome of 526 AD, 247 PD and 573 nonimpaired control individuals (Supplementary Table 1). An initial principal component analysis (PCA) revealed that there was no clustering across all 6,340 proteins (Fig. 1b). We used mutual information (>0.01) to identify *APOE* ε4 proteins. Unlike traditional correlation coefficients, which capture only linear relationships, mutual information can detect both linear and nonlinear associations. This makes it well suited for complex high-dimensional biological data, where relationships between variables may not follow simple patterns. Using this, we identified 229 CSF proteins that were *APOE* ε4 associated in nonimpaired controls (Methods; Supplementary Table 2 and Extended Data Fig. 1a). A subsequent PCA showed that these proteins led to distinct clustering of groups based on *APOE* genotype (Fig. 1c) and the number of *APOE* ε4 alleles (Fig. 1d) independently of disease. This effect was further visualized using a heatmap showing that *APOE* ε4 proteins were upregulated or downregulated based on genotype but not disease (Fig. 1e). Many of these *APOE* ε4 proteins were also identified in our earlier work profiling the CSF proteome of *APOE* ε4 carriers with mild cognitive impairment and AD from the AD Neuroimaging Initiative cohort^17^ further highlighting the robustness and generalizability of our finding.

Using classification and regression trees (CART) modeling, we showed that these 229 CSF proteins were able to reliably (performance metrics > 0.95) predict *APOE* ε4 carriers from noncarriers across AD and PD (Extended Data Table 1 and Fig. 1f). We also found that there were no sex differences in the ability of our identified proteins to distinguish between *APOE* ε4 carriers and noncarriers (Extended Data Table 1 and Fig. 1f). To determine if the *APOE* ε4 signature might be due to differences in amyloid-β levels between carriers and noncarriers, we examined CSF amyloid-β A4 protein levels measured in the SomaScan assay. A Wilcoxon test indicated that there were no significant differences (*P* > 0.05) in CSF amyloid-β A4 protein levels between *APOE* ε4 carriers and noncarriers with AD, PD or nonimpaired controls (Extended Data Fig. 1b).

A functional enrichment analysis in the protein analysis through evolutionary relationships (PANTHER) database^18^ of the CSF *APOE* ε4 proteins revealed significant (false discovery rate (FDR) < 0.05) enrichment for viral processes, apoptosis, rhythmicity, cellular processes, protein phosphorylation and folding and RNA/DNA processes (Fig. 1g and Supplementary Table 3). Given that the most significant enrichment was observed for viral processes, we also performed enrichment analysis for immune pathway-specific processes. Immune-specific Kyoto Encyclopedia of Genes and Genomes (KEGG) pathways revealed that *APOE* ε4 proteins were enriched in numerous infection-related pathways, including hepatitis, herpes, measles, Epstein–Barr virus (EBV), and influenza A. There was also significant enrichment for T cell, B cell and inflammatory signaling cascades, including Toll‑like receptor (TLR), tumor necrosis factor (TNF), interleukin 17 (IL-17), JAK/STAT and nuclear factor-κB (NF-κB; Fig. 1h and Supplementary Table 3). Using single-cell RNA-sequencing data from the Human Protein Atlas^19^, we performed an immune cell subtype enrichment analysis on *APOE* ε4 proteins. Across innate immune cells, *APOE* ε4 proteins were the most enriched in nonclassical and intermediate monocytes. In adaptive immune cells, memory CD8 T cells were the most enriched for *APOE* ε4 proteins, followed by T_regs_ and memory CD4 T cells. In innate-like T cells and lymphoid cells (mixed), both natural killer (NK) cells and γδ T cells showed *APOE* ε4 enrichment (Fig. 1i). Given that we found enrichment for hepatitis KEGG pathways, we also performed a cell-type-specific enrichment analysis in the liver. Across parenchymal cells, *APOE* ε4 proteins were the most enriched in hepatocytes and Kupffer cells, in line with both cell types being the primary producers of the APOE4 proteoform in the liver^20^ (Fig. 1j).

These results show that *APOE* ε4 carriers have a distinct CSF proteomic signature characterized by enriched viral processes and pro-inflammatory immune pathways and cells. We find enrichment across hepatocytes and Kupffer cells in the liver, further implicating APOE4 proteoform synthesis sites in the periphery. This finding may also be reflective of brain-liver signaling and liver responses to neuroinflammation^21^. Notably, these changes were independent of neurodegenerative disease and sex, suggesting that *APOE* ε4 carriers share a common molecular phenotype.

### Plasma proteome profiling reveals a similar *APOE* ε4-specific signature

**Fig. 2:**
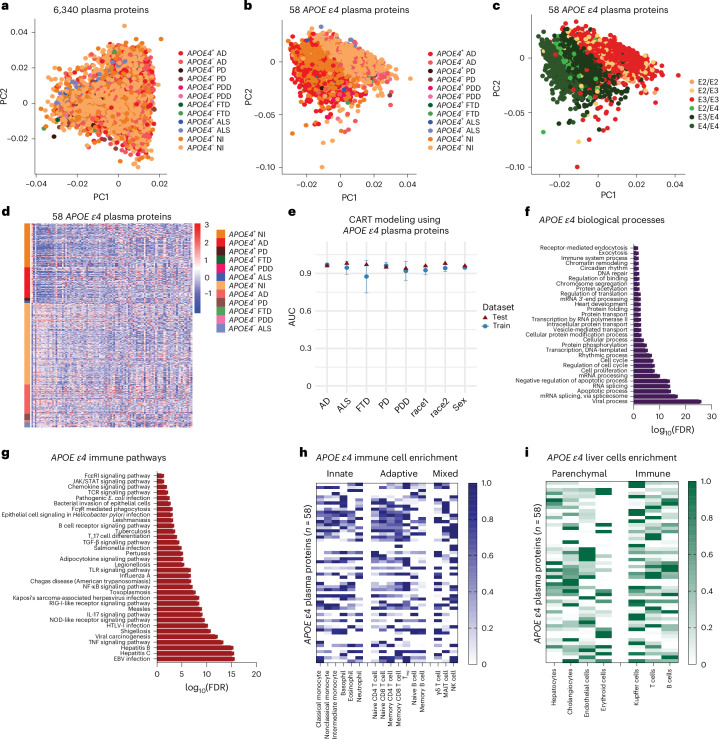
Identification and characterization of the plasma proteome signature in *APOE* ε4 carriers. **a**, PCA of all 6,340 measured plasma proteins showing no clear clustering. **b**, PCA of 58 *APOE* ε4 plasma proteins identified using mutual information that shows clustering based on the presence or absence of *APOE* ε4 allele rather than specific neurodegenerative disease. **c**, PCA of 58 *APOE* ε4 plasma proteins showing that clustering is based on the specific *APOE* genotype and number of *APOE* ε4 alleles (Supplementary Table 1 lists the distribution of *APOE* ε4 cases). **d**, Heatmap visualizing the upregulation (red) and downregulation (blue) of proteins within the *APOE* ε4 plasma proteome signature of 58 proteins that shows distinctions based on the presence or absence of an *APOE* ε4 allele rather than disease. **e**, Supervised machine learning modeling using CART showing mean AUC ± s.d. across fivefold repeated five times. Models were trained and validated on a 70% training dataset and tested using a 30% withheld testing dataset. ‘race1’ refers to American Indian/Alaskan Native individuals and ‘race2’ refers to Black/African American individuals. **f**, Functional enrichment analysis of PANTHER biological processes enriched for *APOE* ε4 plasma proteins showing the most significant (FDR = 1.31 × 10^−^^26^) enrichment for viral processes. **g**, Given the most significant enriched biological process was viral processes, we performed a functional enrichment analysis of KEGG immune-related pathways enriched for *APOE* ε4 plasma proteins. This showed significant (FDR < 0.05) enrichment for immune, infection and pro-inflammatory pathways. **h**, Immune cell-type-specific enrichment analysis of *APOE* ε4 plasma proteins showing involvement across the innate, adaptive and innate-like T cells and lymphoid cells (mixed). **i**, Liver cell-type-specific enrichment analysis of *APOE* ε4 CSF proteins showing involvement across parenchymal and immune cells. Cell-type-specific enrichments are based on single-cell RNA-sequencing data from the Human Protein Atlas^19^. Plot shows min–max scaling of protein-coding transcripts per million for each identified protein in the *APOE* ε4 plasma signature.

**Extended Data Fig. 2:**
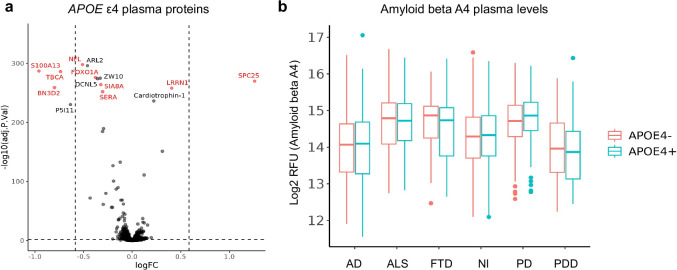
Further characterization of the *APOE* ε4 plasma protein signature. (**a**) Volcano plots showing fold change and adjusted p values for the *APOE* ε4 plasma proteins. Red dots indicate the proteins with the highest mutual information value (>0.1) for reference to the feature selection method used to identify *APOE* ε4 proteins. Differential protein abundance was assessed using linear modeling and empirical Bayes moderation. All tests were two-sided, and p-values were corrected for multiple comparisons using the Benjamini-Hochberg FDR method. Volcano plots display log_2_ fold change on the x-axis and −log_10_-transformed adjusted p-values on the y-axis, with thresholds set at log₂(FC) = ± 0.585 and FDR-adjusted p < 0.05. (**b**) Box plots showing amyloid beta A4 protein plasma levels in relative fluorescent units (RFU) across *APOE* ε4 carriers and non-carriers with AD, ALS, FTD, PDD, PD, and non-impaired controls. The center line represents the median, the bounds of the box indicate the 25th and 75th percentiles (interquartile range; IQR), and the whiskers extend to the minimum and maximum values within 1.5x IQR. Data points outside this range are plotted individually as outliers. N = 2,929 AD, 75 FTD, 169 PDD, 422 PD, 230 ALS, and 6,099 non-impaired control individuals. Abbreviations: AD: Alzheimer’s disease; ALS: amyotrophic lateral sclerosis; FTD: frontotemporal dementia; NI: non-impaired controls; PD: Parkinson’s disease; PDD: Parkinson’s disease dementia.

We next sought to determine whether the *APOE* ε4 CSF proteome changes observed were also reflected in the plasma. Further leveraging the GNPC dataset, we performed plasma proteome profiling of 2,929 AD, 75 FTD, 169 PDD, 422 PD, 230 ALS and 6,099 nonimpaired control individuals with and without an *APOE* ε4 allele (Supplementary Table 1). A PCA of all 6,340 proteins revealed no group clustering (Fig. 2a). Using mutual information, we identified 58 plasma proteins in nonimpaired controls (Supplementary Table 4 and Extended Data Fig. 2a) that were associated with *APOE* genotype (Fig. 2b) and led to clustering based on the number of *APOE* ε4 alleles (Fig. 2c) rather than by neurodegenerative disease. A heatmap also revealed that the 58 *APOE* ε4 plasma changes were upregulated or downregulated based on genotype (Fig. 2d). Two of these proteins, TBCA and LRRN1, were also identified in the serum of healthy *APOE* ε4 centenarians^22^, providing further external validation of our finding and the importance of these proteins in *APOE* ε4 carriers.

CART models using these 58 proteins as predictors showed a strong ability to differentiate between *APOE* ε4 carriers and noncarriers across each of the neurodegenerative disease groups (performance metrics > 0.85; Extended Data Table 2). CART modeling revealed that there were no sex differences in the *APOE* ε4 plasma signature (Extended Data Table 2 and Fig. 2e). We extended this to show that there were no effects of race. Here we trained our models on White individuals and tested them using proteomic data from either Black/African American or American Indian/Alaskan Native individuals. In both cases, our models were reliably able to predict *APOE* ε4 carriers from noncarriers (Extended Data Table 2 and Fig. 2e). We also compared amyloid-β A4 protein levels between *APOE* ε4 carriers and noncarriers across neurodegenerative diseases to determine if the plasma *APOE* ε4 signature was related to amyloid-β pathology. A Wilcoxon test revealed that there were no significant differences in plasma amyloid-β A4 protein between *APOE* ε4 carriers and noncarriers (Extended Data Fig. 2b).

*APOE* ε4 plasma processes were significantly enriched for biological processes, including apoptosis, cellular processes, protein processes and RNA/DNA processes (Supplementary Table 5). As in the CSF, viral processes were the most significantly enriched biological process (Fig. 2f). This was further supported by similar KEGG immune and infection pathway enrichments, including EBV and hepatitis (Fig. 2g). There was also significant enrichment for inflammatory and cytokine signaling pathways, including TNF, IL-17, TLR and NF-κB (Fig. 2g and Supplementary Table 5). Among immune cell subtypes, nonclassical and intermediate monocytes were implicated in *APOE* ε4 proteins, as seen in the CSF. Unlike the CSF, however, basophils were enriched for *APOE* ε4 proteins. Of adaptive immune cell subtypes, plasma *APOE* ε4 proteins were enriched for memory CD8 T cells, T_regs_ and naive CD8 T cells. NK cells and γδ T cells were also associated with *APOE* ε4 (Fig. 2h). In the liver, we found cell-type-specific enrichment primarily for Kupffer cells and T cells and, unlike the CSF, very little enrichment for hepatocytes.

Our results demonstrate that genotype-specific proteomic changes observed in the CSF are also reflected in the plasma of *APOE* ε4 carriers and noncarriers, consistently indicating pro-inflammatory immune dysregulation across multiple pathways and immune cell populations. The *APOE* ε4 proteomic signature remained independent of neurodegenerative disease status and sex, with CART models showing strong predictive power for carrier status. We further showed that this signature generalizes across racial groups, underscoring its robustness and broad applicability. Unlike in the CSF, plasma *APOE* ε4 proteins were not enriched in hepatocytes but were significantly enriched in Kupffer and T cells, suggesting that peripheral immune activation is distinct from central nervous system (CNS) immune-metabolic signaling along the liver-brain axis in *APOE* ε4 carriers.

### Key features of the peripheral immune signature are mirrored in the brains of *APOE* ε4 carriers

**Fig. 3:**
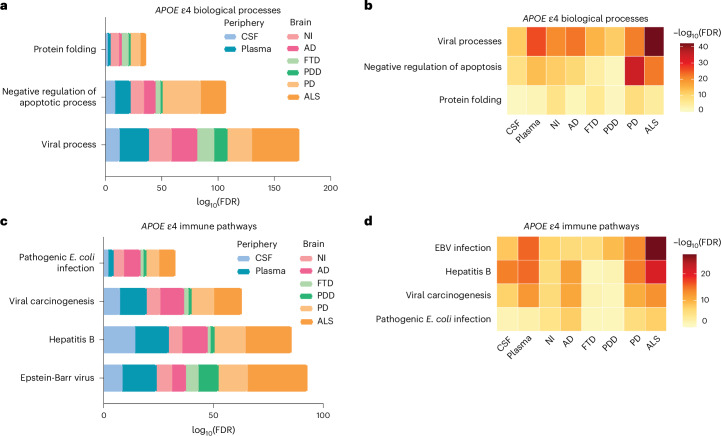
Key overlapping enrichments across the CSF, plasma, and brains of *APOE* ε4 carriers across neurodegenerative diseases. **a**, Bar plot comparing functional enrichment analysis of overlapping significant (FDR < 0.05) PANTHER biological processes enriched for *APOE* ε4 proteins in the CSF, plasma and brain (dlPFC). **b**, Heatmap comparing functional enrichment analysis of overlapping significant (FDR < 0.05) PANTHER biological processes enriched for *APOE* ε4 proteins in the CSF, plasma and brain (dlPFC). **c**, Comparative functional enrichment analysis of significant (FDR < 0.05) KEGG immune-related pathways enriched for *APOE* ε4 proteins in the CSF, plasma and brain (dlPFC). **d**, Comparative functional enrichment analysis of overlapping significant (FDR < 0.05) KEGG immune-related pathways enriched for *APOE* ε4 proteins in the CSF, plasma and brain (dlPFC).

**Extended Data Fig. 3:**
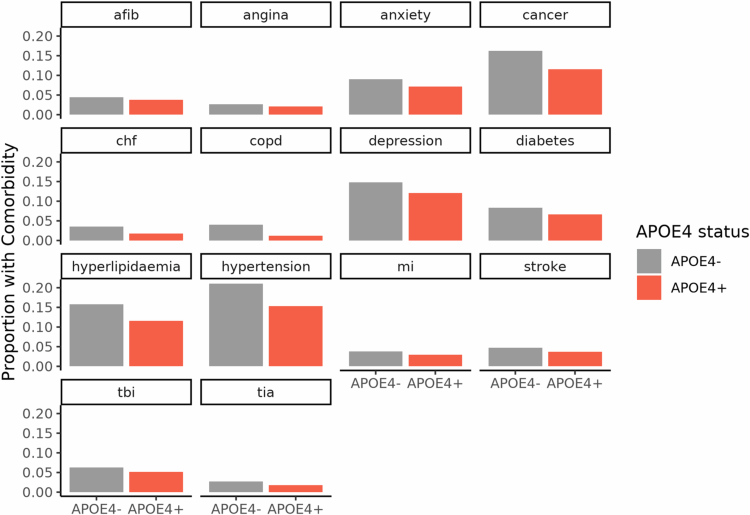
Stratification of comorbidities across *APOE* ε4 carriers and non-carriers. Bar graph representation of the proportion of *APOE* ε4 and non-carriers with comorbidity across the clinical variables included in our correlation network analysis between *APOE* ε4 proteins and demographic, lifestyle, and clinical variables. Only those individuals who said ‘yes’ to having comorbidity are included in the graph (Supplementary Table 1). APOE4+: *APOE* ε4 carrier; APOE4−: *APOE* ε4 non-carrier; afib: atrial fibrillation; chf: congestive heart failure; copd: chronic obstructive pulmonary disease; tbi: traumatic brain injury; tia: transient ischemic attack; mi: myocardial infarction.

We then sought to determine whether the proteomic changes observed in the periphery were reflective of central changes in *APOE* ε4 carriers and to further validate our findings from the GNPC cohort. To do this, we leveraged label-free MS proteomic and postmortem histopathological data from the AMP-AD UPenn Proteomics study for the dlPFC of 49 AD, 31 FTD, 47 PDD, 33 PD, 55 ALS and 47 nonimpaired individual donors (Supplementary Table 6). SomaScan and label-free MS proteomic assays had different coverage of specific proteins; therefore, we focused on confirming enrichment for biological processes and pathways across the CSF, plasma, and dlPFC of carriers and noncarriers. We again used mutual information to identify *APOE* ε4 proteins from the dlPFC within each group independently. Across all neurodegenerative disease groups, we identified 248 *APOE* ε4 proteins (Supplementary Table 7). This was confirmed with PCAs showing clustering based on *APOE* genotype (Extended Data Fig. 3a–l and Supplementary Table 7). Functional enrichment analyses revealed that three of the main biological processes (viral processes, negative regulation of apoptosis, and protein folding) identified in the CSF and plasma were also significantly enriched in the dlPFC of *APOE* ε4 carriers across all neurodegenerative diseases (Fig. 3a,b and Supplementary Table 8). Further, four of the most significantly enriched KEGG immune pathways in the CSF and plasma were also identified in *APOE* ε4 carriers in a disease-independent manner, including EBV, hepatitis B, viral carcinogenesis and pathogenic *Escherichia coli* infection (Fig. 3b,c and Supplementary Table 8).

Previous research suggests that *APOE* ε4 carriers may develop neurodegenerative pathology, including amyloid-β plaques and tau neurofibrillary tangles, even in the absence of symptomatic disease^8,23–26^. We therefore sought to determine if the changes seen in *APOE* ε4 carriers may be due to the presence of *APOE* ε4-driven brain pathology. There was no relationship between *APOE* ε4 and tau or thio-S-positive plaques across nonimpaired controls and disease groups (Supplementary Table 9). In FTD, PDD, and PD, there was also no relationship between *APOE* ε4 and α-synuclein and, in FTD and ALS, no relationship with TDP-43 (Supplementary Table 9). Although none of the neurodegenerative disease groups showed a relationship between *APOE* ε4 and angiopathy, there was a small statistically significant association found in nonimpaired controls (Supplementary Table 9).

Taken together, these findings suggest a relationship between peripheral and central proteomic changes. Both are characterized by pro-inflammatory immune responses, further highlighting that *APOE* ε4 carriers have systemic immune dysregulation. This demonstrates that the major proteome changes seen in the periphery of *APOE* ε4 carriers are indeed reflective of ongoing processes in the brain and that peripheral processes may actively contribute to or even drive changes in the brain proteome. Notably, *APOE* ε4-associated changes are independent of neurodegenerative disease, whether measured simply by diagnosis or by specific pathological changes in the brain.

### Peripheral *APOE* ε4-associated proteins are differentially correlated to clinical variables according to disease

**Fig. 4:**
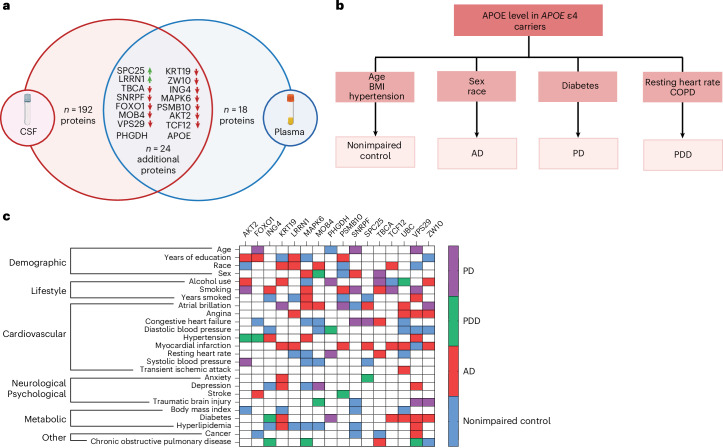
Correlation analyses between *APOE* ε4 CSF and plasma central node proteins and demographic, lifestyle, and clinical variables. **a**, Venn diagram showing the overlapping *APOE* ε4 proteins identified in the CSF and plasma. Of the 40 overlapping proteins, the 16 named proteins represent central protein nodes in the protein–protein interaction network, with more than 20 functional connections. Panel **a** is created with BioRender.com. **b**, Hierarchical tree showing the unique, neurodegenerative disease-specific relationships between APOE and demographic and clinical variables. **c**, Categorical heatmap showing the unique, neurodegenerative-disease-specific relationships between the remaining 15 central node *APOE* ε4 proteins and demographic, lifestyle and clinical (cardiovascular, neurological/psychological, metabolic and other) variables.

**Extended Data Fig. 4:**
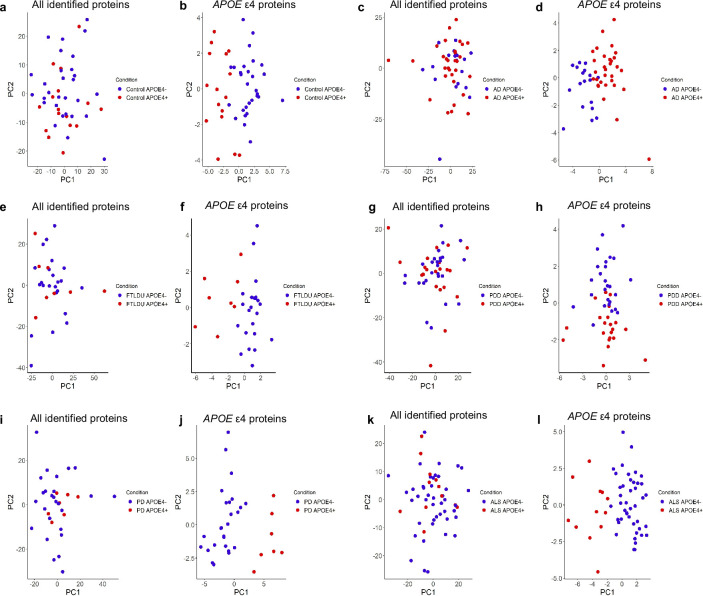
Identification of *APOE* ε4 proteins in the dorsolateral prefrontal cortex of individuals with neurodegenerative diseases and non-impaired controls. *APOE* ε4 proteins identified using label-free MS from the AMP-AD UPenn Proteomics Study cohort and mutual information. (**a**,**c**,**e**,**g**,**i**,**k**) PCA showing all label-free MS measured proteins in each donor group and no clear clustering across *APOE* ε4 carriers and non-carriers. (**b**,**d**,**f**,**h**,**j**,**l**) PCA showing only *APOE* ε4-identified proteins in each donor group, which leads to clustering in each group based on the presence or absence of an *APOE* ε4 allele. AD: Alzheimer’s disease; ALS: amyotrophic lateral sclerosis; FTD: frontotemporal dementia; PD: Parkinson’s disease; PDD: Parkinson’s disease dementia.

Given that *APOE* ε4 carriers across neurodegenerative diseases share major underlying systemic proteomic changes, we sought to better understand additional drivers that may be disease specific. To do this, we leveraged the GNPC dataset and a correlation network analysis to identify the relationship between *APOE* ε4 proteins and demographic, clinical, and lifestyle variables, such as age, blood pressure, and smoking (Supplementary Table 1 and see Extended Data Fig. 4 for a stratification by comorbidities for *APOE* ε4 carriers and noncarriers). Of the 40 proteins that overlapped across the CSF and plasma of *APOE* ε4 carriers, we chose 16 of these that had more than 20 functional connections (Fig. 4a and Supplementary Table 10). These proteins represent central ‘nodes’ in the protein–protein network, suggesting that any changes are more likely to disrupt diverse pathways and functions^27^. Of note, two of the overlapping proteins, APOE and PHGDH, were differentially expressed in opposite directions across the plasma and CSF. Both proteins were upregulated in the CSF but downregulated in the plasma. As we had a higher number of individuals with plasma samples, thereby improving statistical power, the correlation analysis reflects the decreased APOE and PHGDH levels observed in plasma.

We first calculated Spearman’s rank correlation (continuous variables) or correlation ratio (categorical variables) for APOE and demographic, clinical, and lifestyle variables in nonimpaired, AD, PDD and PD *APOE* ε4 carriers. Due to low numbers, we were unable to calculate similar correlations for FTD and ALS. Our analyses revealed unique, neurodegenerative disease-specific significant relationships with APOE (Fig. 4b and Supplementary Table 11). APOE was associated with sex and race in AD, with diabetes in PD, with resting heart rate and chronic obstructive pulmonary disease (COPD) in PDD, and with age, body mass index, and hypertension in nonimpaired controls. A key limitation is the lack of longitudinal data for nonimpaired *APOE* ε4 carriers, who may later develop neurodegenerative disease. Thus, observed associations with clinical variables may reflect preclinical disease. We extended these correlation analyses to the remaining 15 central node *APOE* ε4 proteins. This analysis further identified that *APOE* ε4 central node proteins are significantly correlated (*P* < 0.05) with demographic, clinical, and lifestyle variables in a disease-specific way in *APOE* ε4 carriers (Fig. 4c and Supplementary Table 11).

Together, these findings indicate that although all *APOE* ε4 carriers have the same underlying proteomic signature, specific proteins within this signature are differentially correlated with demographic, lifestyle, and clinical variables. Critically, these correlations were neurodegenerative disease specific.

## Discussion

**Fig. 5:**
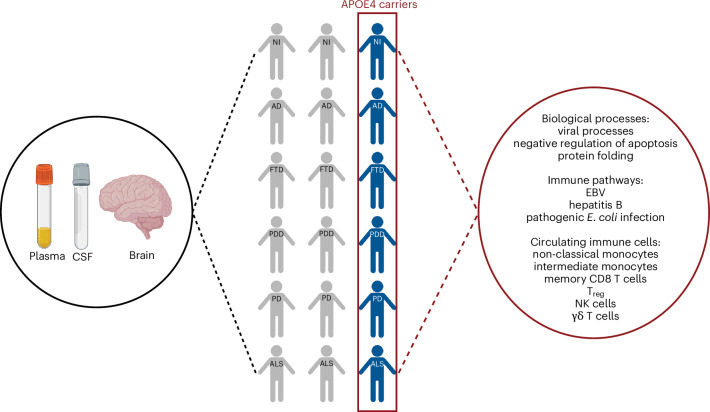
Summary of the study’s findings. *APOE* ε4 carriers across different neurodegenerative diseases share a common systemic proteomic change reflective of pro-inflammatory immune dysregulation. The figure is created with BioRender.com.

The GNPC dataset represents a substantial advancement in neurodegenerative disease research, by providing a real-world clinical proteomic dataset that comprises over 35,000 (11,270 with *APOE* genotype) individuals across AD, FTD, PDD, PD, ALS and normal aging from across more than 20 clinical sites in the US, UK and Europe^28^. This enabled us to ask whether the APOE ε4-associated proteomic signature is shared across multiple neurodegenerative diseases. Our results demonstrate that all *APOE* ε4 carriers, irrespective of neurodegenerative disease, have a unique proteome signature that extends across the plasma and CSF. Unlike prospectively designed cohorts, the GNPC dataset reflects real-world clinical heterogeneity, highlighting the robustness and generalizability of our findings. This signature is associated with pro-inflammatory immune dysregulation and an enrichment for circulating immune cells, including monocytes, memory CD8 and γδ T cells, T_regs_ and NK cells. This molecular phenotype extends to the brains of *APOE* ε4 carriers in a similar disease-independent manner and is not associated with the presence of any disease-specific brain pathology (Fig. 5). Although all *APOE* ε4 carriers have a systemic immune-related proteome signature, we find that the relationships between proteins within this signature are uniquely associated with demographic, lifestyle, and clinical variables in a neurodegenerative disease-specific manner. Notably, this suggests that although the biological changes associated with *APOE* ε4 carriage are essential for neurodegeneration, broadly, interactions with underlying biological vulnerability and the environment may be key for driving the pathogenesis of the specific neurodegenerative disease.

There is evidence that the *APOE* ε4 genotype is a modern-day example of antagonistic pleiotropy. In younger adults, *APOE* ε4 is associated with increased survival and fertility in environments with high levels of infectious disease^29–32^. For example, healthy individuals who are *APOE* ε4 heterozygotes exhibit heightened cytokine release, increased plasma TNF levels, a more pronounced hyperthermic response and an earlier onset of IL-6 production following immune challenge with TLR2/TLR4/TLR5 ligands or lipopolysaccharide^33^. While this immune response protects younger *APOE* ε4 carriers from infectious diseases, prolonged states of inflammation and cytokine release are likely deleterious with age^34,35^. Although this study identified a proteomic signature indicative of a pro-inflammatory phenotype, a limitation is the absence of direct measures of routine inflammatory markers such as C-reactive protein or cytokines. Future studies should incorporate these markers in prospectively designed cohorts to clarify their association with *APOE* ε4.

To date, the biological effects of *APOE* ε4 carriage have largely only been studied in the context of AD. A notable finding of our study is that the pro-inflammatory molecular phenotype associated with *APOE* ε4 extends to individuals with other neurodegenerative diseases, including FTD, PDD, PD and ALS. This raises two key considerations. First, our findings underscore the need to shift focus from the continued identification of genetic risk loci via genome-wide association studies toward functional characterization of established variants^36^. Notably, the absence of a statistically significant association between a variant and a specific disease phenotype does not preclude biological relevance. Despite *APOE* ε4 being overrepresented in the AD cohort, we observe a consistent molecular signature associated with *APOE* ε4 across multiple neurodegenerative diseases, highlighting an underrecognized role for this variant beyond AD that may have been overlooked due to its historically strong link with AD risk. Second, our data support reconceptualizing *APOE* ε4 not only as a disease-specific risk factor but also as a broader susceptibility allele contributing to shared pathogenic mechanisms across neurodegenerative diseases. It remains unclear, however, why *APOE* ε4 is more strongly associated with AD in terms of prevalence despite exhibiting systemic biological effects across neurodegenerative diseases. One possibility is that interactions between *APOE* ε4 and additional age-related, environmental, or comorbid factors may selectively amplify neurodegenerative pathways characteristic of AD. Another possibility is that CNS-specific vulnerabilities or cellular contexts (for example, in hippocampal circuits or dopaminergic neurons) modulate how the *APOE* ε4 inflammatory phenotype manifests clinically. Thus, while *APOE* ε4 confers a shared biological susceptibility, disease expression likely depends on a combination of genetic background, cellular context(s), and lifetime exposures. Large-scale integrative efforts, such as the GNPC, which harmonize data across distinct disease cohorts into unified datasets, provide a powerful framework for advancing this line of inquiry. By enabling cross-disease comparisons, such efforts may help delineate modifiers that influence why some APOE ε4 carriers develop AD while others remain healthy or develop different neurodegenerative diseases. This has important implications for prognosis and risk stratification in midlife individuals who carry *APOE* ε4.

A limitation of our study is the absence of validated biomarkers (for example, CSF p-tau217) to confirm clinical diagnoses, which reflects both the nature of the GNPC dataset and global heterogeneity in clinical practice^28^. However, several features mitigate concerns regarding potential misdiagnosis. First, the *APOE* ε4 signature was derived from nonimpaired individuals. While it is possible that a minority of these nonimpaired individuals harbored asymptomatic pathology (for example, asymptomatic AD), the majority would be free of overt disease, reducing the likelihood of confounding results. Second, the consistent presence of the signature across all *APOE* ε4 carriers, irrespective of clinical diagnosis, supports its generalizability and suggests it reflects a broader *APOE* ε4-related biological phenotype rather than disease-specific changes. Finally, we validate our findings using postmortem brain proteomics and histopathology, where diagnostic certainty is highest. Here *APOE* ε4 status was not associated with hallmark pathologies, including amyloid-β, tau, TDP-43 or α-synuclein, across respective disease groups. This postmortem validation reinforces the robustness of our findings. Future studies would benefit from prospective cohorts incorporating validated CSF or plasma biomarkers to confirm and extend these observations.

An unexpected finding of our study was that plasma neurofilament light (NEFL) levels were lower in APOE ε4 carriers across neurodegenerative diseases, despite NEFL’s growing recognition as a biomarker of neurodegeneration^37^. Prior studies have reported conflicting results—some found no association^38,39^, another reported increased levels in *APOE* ε4 carriers^40^, while others using the SomaScan^41^ and Simoa^42^ assays observed decreased levels, consistent with our findings. These discrepancies may reflect differences in sample size, with studies reporting decreased NEFL levels generally including larger cohorts (~600 to 5,000 participants, and 9,924 in our study). Alternatively, *APOE* ε4-related blood–brain barrier (BBB) or metabolic dysfunction may alter peripheral clearance of NEFL and affect its plasma or CSF concentration. These results raise important questions about the reliability of NEFL as a stand-alone biomarker and underscore the need for mechanistic studies and a shift toward precision biomarkers that integrate genetic and environmental context.

In our study, key peripheral inflammatory states in APOE ε4 carriers were mirrored in the CNS. Notably, brain proteomics performed using label-free MS orthogonally validated findings from SomaScan-based CSF and plasma analyses. While SomaScan aptamer technology offers high-throughput protein quantification, it is relatively insensitive to proteoform diversity, including post-translational modifications^43^, a limitation when examining signaling pathways and protein networks where such modifications are functionally significant. In contrast, MS enables the detection of broader proteoforms, offering an independent assessment of protein abundance and pathway enrichment. The concordance of *APOE* ε4-associated protein signatures and enrichment patterns across plasma, CSF and brain proteomic datasets suggests that our findings are not artifacts of platform-specific biases but likely reflect underlying biology. Supporting this interpretation, we observed enrichment of *APOE* ε4-associated proteins in both hepatocytes and Kupffer cells in the CSF, but only in Kupffer cells and T cells in plasma. This pattern implies that plasma reflects chronic peripheral immune activation, whereas the CSF may capture liver-brain axis-mediated inflammatory signaling in *APOE* ε4 carriers^21^. Further experimental studies are warranted to validate these mechanisms. Prospective cohort studies incorporating cross-platform, multi-tissue analyses from the same individuals will be essential to confirm and expand upon these observations.

While the exact mechanisms underlying the mirroring of peripheral and central pro-inflammatory states remain unclear, they may involve interactions between pro-inflammatory peripheral immune cells and the BBB. A recent study of individuals with long COVID-19 demonstrated that hyperactive peripheral blood mononuclear cells adhere to the endothelial cells of the BBB, driving inflammation, degradation, and symptoms of brain fog^44^. In support of this, cognitively healthy *APOE* ε4 carriers exhibit early markers of BBB dysfunction in the hippocampus and medial temporal lobe^45^. As the BBB becomes increasingly compromised, it allows the infiltration of blood-derived proteins and pro-inflammatory immune cells into the brain, contributing to exacerbated neuroinflammation and neurodegeneration^46^. In our study, we identify enriched pro-inflammatory immune cell subpopulations, including nonclassical and intermediate monocytes^47,48^, memory CD8^+^ T cells^49^, γδ T cells^50^ and NK cells^51,52^. These immune cell types may represent peripheral blood mononuclear cells that interact with the BBB to promote neurodegeneration in *APOE* ε4 carriers. Given that *APOE* ε4 is linked to a shared molecular phenotype across neurodegenerative diseases, this hypothesis is consistent with current understanding. Healthy, nonimpaired *APOE* ε4 carriers have a systemic pro-inflammatory phenotype^17^ (and as we have shown here), early signs of BBB disruption^45^, alterations in brain activity and connectivity^53,54^, and sleep disturbances^55^, all of which are known to be risk factors for neurodegenerative diseases, broadly^56–58^. Further supporting this is the recent evidence that vaccinations protect against dementia^59,60^, including the shingles vaccines Zostavax^61^ and Shingrix^62^. Although none of these studies explicitly examined the interactive effects between vaccination status and *APOE* ε4 genotype, the fact that more than 60% of individuals with dementia are *APOE* ε4 carriers^63^ suggests that *APOE* ε4 may represent a critical modifier of the observed associations, warranting further investigation. Future studies in human disease-relevant models, such as patient stem cell-derived organoids, are needed to further elucidate these mechanisms.

If all *APOE* ε4 carriers share a common underlying biological vulnerability to neurodegeneration, an important question is what determines which specific neurodegenerative disease they develop. This likely reflects complex interactions between genetic risk and environmental exposures^64^. In our study, proteins within the *APOE* ε4 signature were differentially correlated with demographic, lifestyle, and clinical variables, in a neurodegenerative disease-specific manner. This suggests that genotype–environment interactions may modulate disease trajectories in *APOE* ε4 carriers. While limited by the variables consistently collected across the GNPC’s global real-world cohorts, our findings highlight meaningful associations that warrant further investigation. Future prospective studies should incorporate more detailed environmental and behavioral measures, such as physical activity, diet, sleep, substance use and immunization history, to better characterize modifiers of neurodegenerative disease risk in *APOE* ε4 carriers. Given the cross-sectional nature of our study, we cannot infer causality or perform mediation analyses, as clinical variables like hypertension may have arisen after disease onset. One plausible mechanism is that *APOE* ε4 carriers exhibit heightened inflammatory responses to environmental or pathological stressors, including comorbidities such as hypercholesterolemia and ischemic heart disease, to which they are predisposed^65–67^. This suggests that the pro-inflammatory phenotype of *APOE* ε4 carriers may be, at least in part, independent of cardiovascular comorbidities. Our findings further support this interpretation. Our PANTHER biological processes enrichment analyses of the *APOE* ε4 CSF proteome showed no significant enrichment of cardiovascular-related processes. In the plasma, we found only a single enrichment for heart development; however, this was modest relative to the top enrichment of viral processes. These results suggest that additional environmental or pathological insults may act synergistically with a genetically primed pro-inflammatory state to drive neurodegeneration in *APOE* ε4 carriers. Another interpretation of our data, however, is that individuals with pre-existing neurodegenerative diseases are more likely to develop comorbidities^68,69^, such as hypertension, which may be a consequence of neurodegenerative processes rather than a precursory event. Longitudinal studies tracking *APOE* ε4 carriers over time will be essential to disentangle causal pathways and advance personalized prevention strategies.

Neurodegenerative disease risk is reflective of a complex polygenic architecture and co-occurring variants in genes, such as *TREM2*, *MAPT*, *GRN*, *GBA* and *LRRK2*, may modulate or interact with *APOE* ε4-associated molecular pathways. For example, joint carriage of *APOE* ε4 and *MAPT* mutations resulted in a significantly lower age of FTD onset^6^. *APOE* ε4 and *GBA* and *LRRK2* carriers also have the highest risk of PDD^13^. Genome-wide genetic data beyond *APOE* genotype, however, are not currently available within the GNPC cohort and therefore could not be included in the present study. Despite this, a key strength of our study is the demonstration that a well-established risk variant for one neurodegenerative disease (*APOE* ε4 in AD) also exerts a conserved molecularsignature across multiple neurodegenerative conditions. These findings provide proteomic evidence that a single risk variant is implicated across the neurodegenerative disease spectrum. This indicates the need for a conceptual shift in the field, moving away from viewing genetic risk variants as disease specific and toward recognizing their potential as shared, pleiotropic modifiers of neurodegenerative vulnerability. Future work, therefore, would greatly benefit from a focus on the role of other genetic variants across the neurodegenerative disease spectrum, even if they are enriched in only one disease.

Overall, our study provides a conceptual and translational advance in understanding *APOE* ε4-mediated risk for neurodegeneration. We identify a conserved, systemic pro-inflammatory immune proteomic signature associated with *APOE* ε4 across plasma, CSF and brain, irrespective of neurodegenerative disease or pathology. Notably, this signature is also present in asymptomatic individuals, suggesting it precedes clinical symptom onset and reflects an intrinsic biological vulnerability. The ability to detect this signature in plasma, a minimally invasive and clinically accessible biofluid, also offers promising opportunities for blood-based precision biomarkers to identify individuals at risk before symptom onset. Together, these findings demonstrate that *APOE* ε4 confers an intrinsic biological vulnerability characterized by a chronic pro-inflammatory immune phenotype that is independent of downstream disease processes. While this chronic immune activation may predispose *APOE* ε4 carriers to neurodegeneration, it is unlikely to be sufficient alone to drive the development of neurodegenerative disease. Instead, the transition to clinical disease likely involves complex interactions between genetic susceptibility and modifiable environmental, lifestyle, and clinical factors. By linking the *APOE* ε4 signature to such factors, our findings provide actionable insights for early intervention and prevention. They also establish a roadmap for future exploration of gene–gene and gene–environment interactions, including polygenic and epistatic effects. More broadly, our findings reframe *APOE* ε4 as a pleiotropic immune modulator rather than an AD-specific gene, underscoring the need for cross-disease, genetically informed models of risk stratification and therapy. Finally, this work lays the foundation for future wet laboratory-based mechanistic studies to elucidate how *APOE* ε4 shapes immune function and neuroinflammatory signaling, a critical step toward developing targeted and individualized immunotherapies for early and preclinical disease.

## Methods

### Participants

#### GNPC cohort

The GNPC cohort represents the largest collection of SomaScan proteomic data for individuals with neurodegenerative diseases and nonimpaired controls sourced from study sites across the USA, UK and Europe. In the present study, we included 11,270 individuals from the GNPC cohort, with 6,672 nonimpaired controls, 3,455 AD, 75 FTD, 169 PDD, 669 PD and 230 ALS individuals. Of these, 4,325 individuals were *APOE* ε4 carriers (either heterozygous or homozygous) and 6,945 were non-*APOE* ε4 carriers. All included individuals in the study provided either CSF or plasma samples, but none provided both. Individuals were diagnosed based on diagnostic criteria from each research group, as described in the GNPC cohort summary paper^1^. Cognitive impairment for AD and PDD patients was further assessed using a clinical dementia rating score of ≥1, Mini-Mental State Exam score of ≤24 and/or Montreal Cognitive Assessment score of ≤23. Most individuals with AD had cognitive impairment scores indicative of mild or moderate AD. We excluded individuals with mild cognitive impairment in the current study due to its heterogeneity as a clinical diagnosis, and because it does not necessarily reflect neurodegenerative pathology. CSF or plasma samples were collected from all participants at a single timepoint along with demographic and clinical variables (Supplementary Table 1). Participants from each of the included study sites in the GNPC cohort provided written informed consent and studies were approved by the relevant institution’s ethics committee^28^.

#### AMP-AD UPenn Proteomics Study cohort

The AMP-AD UPenn Proteomics Study is a cohort of autopsy-collected samples from the dlPFC of 49 AD, 31 FTD (with TDP-43 inclusions), 47 PDD, 33 PD, 55 ALS and 47 nonimpaired individual donors from the University of Pennsylvania School of Medicine Brain Bank (https://www.synapse.org/Synapse:syn21438414). Individual diagnoses were confirmed through postmortem neuropathological evaluation for neuritic plaque distribution according to the Consortium to Establish a Registry for AD criteria^70^ and neurofibrillary tangle pathology according to the Braak staging system^71^. Other neuropathologic assessments, including for α-synuclein, TDP-43, gliosis and angiopathy, were made in line with established criteria and guidelines^72,73^. Demographic variables for the cohort are listed in Supplementary Table 6. Human postmortem tissues were acquired under proper institutional review board protocols.

### Proteomics

#### GNPC cohort

CSF and plasma proteomics in the GNPC cohort were performed using the SomaScan v4.1 assay, which measures approximately 7,000 proteins^74,75^. This technology uses aptamer-based approaches, specifically slow off-rate modified aptamers, which incorporate chemically modified nucleotides that bind with high specificity and affinity to target proteins^74^. SomaLogic provides ‘raw’ data that has been standardized, normalized, and calibrated, including using adaptive normalization by maximum likelihood. Protein measurements are provided in relative fluorescent units. Aptamers were mapped to Uniprot before being included in the GNPC cohort dataset. Details on the creation and harmonization of this dataset are described elsewhere^28^. Before our own analyses, we log_2_ transformed and standardized training and testing datasets separately.

#### AMP-AD UPenn Proteomics Study cohort

Proteins from postmortem brain homogenates from the dlPFC were measured using label-free MS on a Q-Exactive Plus mass spectrometer as previously described^76^. Proteins included in this study were identified using MaxQuant’ MaxLFQ (label-free quantification) algorithm. Normalized label-free quantitation protein intensities were extracted from the proteinGroups.txt MaxQuant output file using a custom R script (LoaderAndBatchCorrection-UPenn354cases.R)^76^. To account for missing protein quantitation values in the dataset, we imputed values using the median for proteins with ≤20% missing values and excluded those proteins with ≥20% missing values.

### Statistical analyses

#### Feature selection

We identified *APOE* ε4-associated proteins using mutual information, a statistical measure that quantifies the amount of information one variable provides about another^77^. In the context of our study, it measures how informative each protein is for distinguishing *APOE* ε4 carriers from noncarriers. A mutual information score of zero indicates no association, while higher values indicate greater dependency between a protein and *APOE* ε4 status. We used a cutoff of ≥0.01 to indicate proteins that were likely to be associated with *APOE* ε4 carrier status. For CSF and plasma, we used nonimpaired controls to identify *APOE* ε4-associated proteins. This ensured that we were identifying proteins in only those individuals who are not symptomatic for any one neurodegenerative disease. Appropriate feature selection (identification of proteins specifically associated with *APOE* ε4) was further confirmed using PCA and heatmap. In the brain, we used mutual information to identify *APOE* ε4 proteins specific to each group of individuals. PCA was then used to confirm that within each group, *APOE* ε4-identified proteins clustered based on *APOE* ε4 carrier status. For visualization of differential protein abundance, volcano plots were constructed using log_2_ (fold change(FC)) on the *x* axis and −log_10_-transformed adjusted *P* values on the *y* axis. Plots show fold change and significance thresholds that were applied at log_2_(FC) = ± 0.585 (corresponding to a 1.5-fold change) and FDR-adjusted *P* < 0.05, respectively. Mutual information was calculated in R (v4.4.1) using the package ‘FSelectorRcpp’^78^ and PCA plots and heatmaps were made using ‘ggplot2’^79^ and ‘pheatmap’, respectively.

#### CART

The generalizability of our identified *APOE* ε4 proteins across different neurodegenerative diseases was tested. We performed this analysis using supervised machine learning with CART via the ‘caret’ package in R (v4.4.1). For all CART models, we first split the dataset into a 70% training and validation dataset and a 30% withheld (unseen) testing dataset. Model training and evaluation were conducted using ‘caret’ with a fivefold cross-validation procedure repeated five times. Where classes (groups) were imbalanced, we addressed this by using upsampling. Model performance was assessed using the 30% unseen dataset and several metrics, including sensitivity, specificity, positive predictive value, negative predictive value and area under the curve. To identify potential sex-specific differences in the generalizability of *APOE* ε4 proteins, we trained and tested the CART models on a mixed sample of male and female *APOE* ε4 carriers and noncarriers. To assess any race-specific effects in plasma, we trained the CART models on White *APOE* ε4 carriers and noncarriers and then tested them using only individuals of another race (Black/African American or American Indian/Alaskan Native).

#### Linear regression

To identify a potential relationship between *APOE* ε4 carrier status and postmortem histopathology in the dlPFC, we used a linear regression. Postmortem histopathology data for all donors included tau, thio-S-positive plaques, and angiopathy. Additional measures for AD and nonimpaired donors included antibody-positive plaques, Thal amyloid score, and gliosis. FTD, PDD and PD donors also had postmortem histopathological assessments for α-synuclein. FTD and ALS donors included TDP-43 assessments.

#### Correlation network analysis

To determine a potential relationship between overlapping CSF and plasma proteins identified as being *APOE* ε4-specific and demographic, clinical and lifestyle variables, we performed a correlation network analysis. Protein levels were based on those measured in the plasma, although only overlapping *APOE* ε4 identified across both CSF and plasma were included. Before computing any correlations, rows with missing values or encoded as an indeterminate response (for example, ‘unsure’, coded as −1 or NA) were excluded on a pairwise basis. This ensured that each test was conducted only on complete and valid data. For associations between two continuous variables, we used Spearman’s rank correlation coefficient (*ρ*). For associations between categorical variables and continuous protein expression values, we employed a correlation ratio (*η*^2^), which measures the proportion of variance in the continuous variable explained by the grouping variable. This was calculated by fitting a linear model with the continuous variable as the outcome and the categorical variable as the predictor. The coefficient of determination (*R*^2^) from the model was interpreted as *η*^2^. The statistical significance of the association was determined using ANOVA (*P* < 0.05). Categorical variables included binary indicators (for example, presence/absence of comorbidities) as well as variables with more than two levels (for example, ‘yes’, ‘no’, ‘unsure’). All categorical variables were explicitly converted to factors, and associations were computed only where at least two factor levels were present in the subset of complete cases. The correlation network analysis figure was created using GraphPad Prism (v10.0.0 for Windows).

#### Enrichment analyses

Protein–protein enrichment analyses to assess the functions enriched for *APOE* ε4 proteins were done using NetworkAnalyst (v3.0)^80–82^. Here generic protein–protein interactions were identified using a first-order network from the International Molecular Exchange Consortium interactome database^83^ and InnateDB^84^. Network enrichment for biological processes was performed using the PANTHER classification system^18^ and KEGG pathways^85^. Significance was determined by an FDR of >0.05.

Immune cell-type-specific enrichment analyses for immune and liver cells were done using single-cell RNA-sequencing data from the Human Protein Atlas (https://www.proteinatlas.org/, v23; Ensembl v109)^19^. For each *APOE* ε4 protein, we identified the corresponding protein-coding transcripts per million counts. We then normalized the expression for each cell type using min–max scaling and used heatmaps to visually represent these enrichments. Enrichment bar graphs and heatmaps were created using GraphPad Prism (v10.0.0 for Windows).

### Reporting summary

Further information on research design is available in the Nature Portfolio Reporting Summary linked to this article.

## Online content

Any methods, additional references, Nature Portfolio reporting summaries, source data, extended data, supplementary information, acknowledgements, peer review information; details of author contributions and competing interests; and statements of data and code availability are available at 10.1038/s41591-025-03835-z.

## Supplementary information

Supplementary Information List of GNPC members.

Reporting Summary

Supplementary Tables 1–11 Supplementary Tables 1–11.

## Figures and Tables

**Extended Data Table 1:**
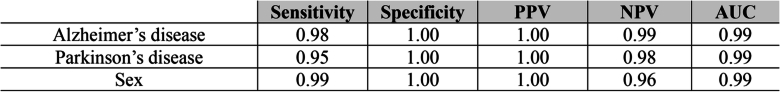
Performance metrics of CART models for predicting *APOE* ε4 carriers from non-carriers in AD, PD, and by sex using *APOE* ε4 CSF proteins (n = 229) as predictive features

AUC: area under the curve; NPV: negative predictive value; PPV: positive predictive value.

**Extended Data Table 2:**
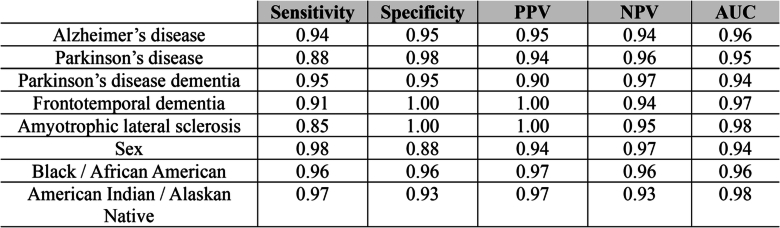
Performance metrics of CART models for predicting *APOE* ε4 carriers from non-carriers in AD, PD, and by sex using *APOE* ε4 plasma proteins (n = 58) as predictive features

AUC: area under the curve; NPV: negative predictive value; PPV: positive predictive value.

## Data Availability

The harmonized GNPC data used to generate these findings was provided to Consortium Members in June 2024 and will be made available for public request by the AD Data Initiative by 15 July 2025. Members of the global research community will be able to access the metadata and place a data use request via the AD Discovery Portal (https://discover.alzheimersdata.org/). Access is contingent on adherence to the GNPC Data Use Agreement and the Publication Policies. The AMP-AD UPenn Proteomics Study data is available through the AD Knowledge Portal (https://adknowledgeportal.synapse.org/). Researchers who wish to access this controlled dataset are required to submit a Data Use Agreement. More information can be found here: https://adknowledgeportal.synapse.org/Data%20Access.

## Code availability

All code used for analyzing the proteomic and clinical data are available at https://github.com/Art83/gnpc_apoe.
