# Supplementary material for: APOE ε4 carriers share immune-related proteomic changes across neurodegenerative diseases
Source: Nat Med. 2025 Jul 15;31(8):2590–601. doi: 10.1038/s41591-025-03835-z (PMC12353839; doi:10.1038/s41591-025-03835-z)
Supplement: Supplementary file 1 — List of GNPC members. [file 41591_2025_3835_MOESM1_ESM.pdf]

nature medicine

Supplementary information https://doi.org/10.1038/s41591-025-03835-z

APOE ε4 carriers share immune-related proteomic changes across neurodegenerative diseases

In the format provided by the authors and unedited

GNPC V1 Full Membership List and Affiliations

- Gamal Abdel-Azim, Johnson & Johnson, Spring House, USA
- Charles H Adler, Mayo Clinic Arizona, Scottsdale, Arizona, USA
- Muhammad Ali, Washington University School of Medicine, Department of Psychiatry, St. Louis, 63110, MO, USA.; NeuroGenomics and Informatics Center, Washington University School of Medicine, St. Louis, 63110, MO, USA.
- Lijun An, Department of Clinical Sciences Malmö, SciLifeLab, Lund University, Lund, Sweden
- Federica Anastasi, Barcelonaβeta Brain Research Center (BBRC), Pasqual Maragall Foundation, Barcelona, Spain; Hospital del Mar Research Institute, Barcelona, Spain; Centre for Genomic Regulation (CRG), Barcelona Institute of Science and Technology (BIST), Barcelona, Spain
- Alireza Atri, Banner Sun Health Research Institute, Sun City, Arizona, USA
- Thomas G Beach, Banner Sun Health Research Institute, Sun City, Arizona, USA
- Graham Bearden, Alzheimer's Disease Data Initiative, Kirkland, WA
- David Bennett, Rush Alzheimer's Disease Center, Department of Neurological Sciences, Chicago, IL, USA
- James D. Berry, Sean M. Healey and AMG Center for ALS, Neurology
- Alexa Pichet Binette, Clinical Memory Research Unit, Department of Clinical Sciences Malmö, Lund University, Lund, Sweden; Department of Physiology and Pharmacology, Université de Montréal, Montreal, Quebec, Canada; Montreal Geriatrics Institute Research Center, Montreal, Quebec, Canada
- Merce Boada, Ace Alzheimer Center Barcelona, Universitat Internacional de Catalunya, 08029 Barcelona, Spain; Biomedical Research Networking Centre in Neurodegenerative Diseases (CIBERNED), National Institute of Health Carlos III, 28029 Madrid, Spain
- Merle Bode, Hertie Institute for Clinical Brain Research, Neurodegenerative Diseases, Tübingen; German Center of Neurodegenerative Diseases , Department of Neurodegenerative Diseases, Tübingen
- Bradley Boeve, Mayo Clinic, Neurology Department, Rochester, MN
- Niranjan Bose, Gates Ventures, Seattle, WA
- Veronica Bot, Stanford University, The Phil and Penny Knight Initiative for Brain Resilience, Stanford, CA, USA; Stanford University, Wu Tsai Neurosciences Institute, Stanford, CA, USA; Stanford University, Graduate Program in Biomedical Engineering, Stanford, CA, USA
- Hillary Bounds, Gates Ventures, Seattle, WA
- Adam L. Boxer, University of California, San Francisco, Neurology Department, San Francisco, CA
- Martin Bringmann, Johnson & Johnson, Spring House, USA
- Alfredo Cabrera-Socorro, Johnson & Johnson, NS TA, Beerse, Belgium
- Amanda Fernandez Cano, Ace Alzheimer Center Barcelona, Universitat Internacional de Catalunya, 08029 Barcelona, Spain; Biomedical Research Networking Centre in Neurodegenerative Diseases (CIBERNED), National Institute of Health Carlos III, 28029 Madrid, Spain

- Kaitlin B. Casaletto, University of California, San Francisco, Neurology Department, San Francisco, CA
- Richard J Caselli, Mayo Clinic Arizona, Scottsdale, Arizona, USA
- Yike Chen, Washington University School of Medicine, Department of Psychiatry, St. Louis, 63110, MO, USA.; NeuroGenomics and Informatics Center, Washington University School of Medicine, St. Louis, 63110, MO, USA.
- Matthew H.S. Clement, Alzheimer's Disease Data Initiative, Kirkland, WA
- Carlos Cruchaga, Washington University School of Medicine, Department of Psychiatry, St. Louis, 63110, MO, USA.; NeuroGenomics and Informatics Center, Washington University School of Medicine, St. Louis, 63110, MO, USA.; Washington University School of Medicine, Department of Neurology, St. Louis, 63110, MO, USA.
- Jeff Dage, Indiana Alzheimer's Disease Research Center, Indianapolis, IN; Indiana University School of Medicine, Department of Neurology, Indianapolis, IN
- Eric B. Dammer, Emory University School of Medicine, Atlanta, GA, USA; Emory University School of Medicine, Department of Biochemistry, Atlanta, GA, USA
- Sterre de Boer, Alzheimer Center Amsterdam, Neurology, Amsterdam UMC, Amsterdam, the Netherlands; Amsterdam Neuroscience, Amsterdam, the Netherlands
- Niels De Meirleir, Johnson & Johnson, NS TA, Beerse, Belgium
- Marta del Campo Milan, Barcelonaβeta Brain Research Center (BBRC), Pasqual Maragall Foundation, Barcelona, Spain; Hospital del Mar Research Institute, Barcelona, Spain
- Daisy Ding, Stanford University, The Phil and Penny Knight Initiative for Brain Resilience, Stanford, CA, USA; Stanford University, Wu Tsai Neurosciences Institute, Stanford, CA, USA; Stanford University, Graduate Program in Biomedical Engineering, Stanford, CA, USA
- Duc Duong, Emory University School of Medicine, Atlanta, GA, USA; Emory University School of Medicine, Department of Biochemistry, Atlanta, GA, USA
- Amelia Farinas, Stanford University, Graduate Program in Neuroscience, Stanford, CA, USA; Stanford University, The Phil and Penny Knight Initiative for Brain Resilience, Stanford, CA, USA; Stanford University, Wu Tsai Neurosciences Institute, Stanford, CA, USA
- Maria Victoria Fernandez, Ace Alzheimer Center Barcelona, Universitat Internacional de Catalunya, 08029 Barcelona, Spain
- Luigi Ferrucci, NIH/NIA, Translational Gerontology Branch, National Institute on Aging
- Lawrence Fourgeaud, Johnson & Johnson, NS TA, La Jolla, USA
- Mark Frasier, Michael J. Fox Foundation, New York, NY, USA
- Raquel Puerta Fuentes, Ace Alzheimer Center Barcelona, Universitat Internacional de Catalunya, 08029 Barcelona, Spain; PhD Program in Biotecnology, Faculty of Pharmacy and Food Sciences, University of Barcelona, 08028 Barcelona, Spain
- Jordan Fuller, Gates Ventures, Seattle, WA
- Su Gao, Indiana Alzheimer's Disease Research Center, Indianapolis, IN; Indiana University School of Medicine, Department of Biostatistics & Health Data Science, Indianapolis, IN
- John Gibbons, Rush Alzheimer's Disease Center, Department of Neurological Sciences, Chicago, IL, USA
- Pablo Garcia Gonzalez, Ace Alzheimer Center Barcelona, Universitat Internacional de Catalunya, 08029 Barcelona, Spain; Biomedical Research Networking Centre in

Neurodegenerative Diseases (CIBERNED), National Institute of Health Carlos III, 28029 Madrid, Spain

- Oskar Hansson, Clinical Memory Research Unit, Department of Clinical Sciences Malmö, Lund University, Lund, Sweden
- Gyujin Heo, Washington University School of Medicine, Department of Psychiatry, St. Louis, 63110, MO, USA.; NeuroGenomics and Informatics Center, Washington University School of Medicine, St. Louis, 63110, MO, USA.
- Hilary Heuer, University of California, San Francisco, Neurology Department, San Francisco, CA
- Timothy J. Hohman, Vanderbilt Memory & Alzheimer's Disease, Department Neurology, Vanderbilt University Medical Center, Nashville, TN ; Vanderbilt Genetics Institute, Vanderbilt Medical Center, Nashville, TN, USA
- Liping Hou, Johnson & Johnson, Spring House, USA
- Yen-Ning Huang, Indiana Alzheimer's Disease Research Center, Indianapolis, IN; Indiana University School of Medicine, Department of Radiology & Imaging Sciences, Indianapolis, IN
- Alina Isakova, Stanford University, The Phil and Penny Knight Initiative for Brain Resilience, Stanford, CA, USA
- Clifford R. Jack, Jr, Mayo Clinic, Radiology
- Mika Kivimaki, University College London, UCL Brain Sciences, London, UK; University of Helsinki, Clinicum, Helsinki, Finland
- Emily Kogan, Johnson & Johnson, JRD DSDH, Cambridge, USA
- Roxanna Korologou-Linden, Ageing & Epidemiology (AGE) Research Unit, School of Public Health, Imperial College London, UK
- Jessica B Langbaum, Banner Alzheimer's Institute, Phoenix, Arizona, USA
- Argentina Lario-Lago, University of California, San Francisco, Neurology Department, San Francisco, CA
- Agustin Ruiz Laza, Ace Alzheimer Center Barcelona, Universitat Internacional de Catalunya, 08029 Barcelona, Spain; Biomedical Research Networking Centre in Neurodegenerative Diseases (CIBERNED), National Institute of Health Carlos III, 28029 Madrid, Spain; Glenn Biggs Institute for Alzheimer's & Neurodegenerative Diseases and Department of Microbiology, Immunology and Molecular Genetics, Long School of Medicine, University of Texas Health Science Center, San Antonio, TX 77204, USA
- Allan I. Levey, Emory University School of Medicine, Atlanta, GA, USA; Emory University School of Medicine, Department of Neurology, Atlanta, GA, USA
- Shuwei Li, Johnson & Johnson, Spring House, USA
- Inga Liepelt-Scarfone, Hertie Institute for Clinical Brain Research, Neurodegenerative Diseases, Tübingen; German Center of Neurodegenerative Diseases , Department of Neurodegenerative Diseases, Tübingen; IB Hochschule für Gesundheit und Soziales , Standort Stuttgart
- Shiwei Liu, Indiana Alzheimer's Disease Research Center, Indianapolis, IN; Indiana University School of Medicine, Department of Radiology & Imaging Sciences, Indianapolis, IN

- Menghan Liu, Washington University School of Medicine, Department of Psychiatry, St. Louis, 63110, MO, USA.; NeuroGenomics and Informatics Center, Washington University School of Medicine, St. Louis, 63110, MO, USA.
- Simon Lovestone, Johnson & Johnson, London, UK
- Lina Lu, Clinical Memory Research Unit, Department of Clinical Sciences Malmö, Lund University, Lund, Sweden
- Marta Marquie, Ace Alzheimer Center Barcelona, Universitat Internacional de Catalunya, 08029 Barcelona, Spain; Biomedical Research Networking Centre in Neurodegenerative Diseases (CIBERNED), National Institute of Health Carlos III, 28029 Madrid, Spain
- Niklas Mattsson-Carlgren, Clinical Memory Research Unit, Department of Clinical Sciences Malmö, Lund University, Lund, Sweden; Memory Clinic, Skåne University Hospital, Malmö, Sweden
- Caitlin P. McHugh, Alzheimer's Disease Data Initiative, Kirkland, WA
- Martine Meyer, Johnson & Johnson, NS TA
- Lefkos T. Middelton, Ageing & Epidemiology (AGE) Research Unit, School of Public Health, Imperial College London, UK
- Silke Miller, Johnson & Johnson, NS TA, La Jolla, USA
- Elizabeth Mlynarski, Johnson & Johnson, JRD DSDH, Spring House, USA
- Diederik Moechars, Johnson & Johnson, NS TA, Beerse, Belgium
- Patricia Moran-Losada, Stanford University, The Phil and Penny Knight Initiative for Brain Resilience, Stanford, CA, USA; Stanford University, Wu Tsai Neurosciences Institute, Stanford, CA, USA; Stanford University School of Medicine, Department of Neurology and Neurological Sciences, Stanford, CA, USA
- Kwangsik Nho, Indiana Alzheimer's Disease Research Center, Indianapolis, IN; Indiana University School of Medicine, Department of Radiology & Imaging Sciences, Indianapolis, IN
- Hamilton Oh, Stanford University, The Phil and Penny Knight Initiative for Brain Resilience, Stanford, CA, USA; Stanford University, Wu Tsai Neurosciences Institute, Stanford, CA, USA; Mount Sinai, Icahn School of Medicine at Mount Sinai, New York NY, USA
- Paige Opsahl, Gates Ventures, Seattle, WA
- Tamina Park, Indiana Alzheimer's Disease Research Center, Indianapolis, IN; Indiana University School of Medicine, Department of Radiology & Imaging Sciences, Indianapolis, IN
- Ronald C. Petersen, Mayo Clinic, Neurology, Rochester, MN
- Mukta Phatak, Alzheimer's Disease Data Initiative, Kirkland, WA
- Joni Lindbohm, MD, PhD, University College London, UCL Brain Sciences, London, UK; University of Helsinki, Clinicum, Helsinki, Finland
- Joseph Pick, Johnson & Johnson, Spring House, USA
- Yolande AL Pijnenburg, Alzheimer Center Amsterdam, Neurology Department, Amsterdam, the Netherlands; Amsterdam Neuroscience, Amsterdam, the Netherlands
- Michael Price, Michael J. Fox Foundation, New York, NY, USA
- Eric M Reiman, Banner Alzheimer's Institute, Phoenix, Arizona, USA

- Shannon Risacher, Indiana Alzheimer's Disease Research Center, Indianapolis, IN; Indiana University School of Medicine, Department of Radiology & Imaging Sciences, Indianapolis, IN
- Oliver Robinson, Ageing & Epidemiology (AGE) Research Unit, School of Public Health, Imperial College London, UK; Department of Epidemiology and Biostatistics, School of Public Health, Imperial College London, UK
- Julio C. Rojas, University of California, San Francisco, Neurology Department, San Francisco, CA
- Howard J. Rosen, University of California, San Francisco, Neurology Department, San Francisco, CA
- Rowan Saloner, University of California, San Francisco, Neurology Department, San Francisco, CA
- Tamsin Sargood, Johnson & Johnson, Global Development, UK
- Andrew J. Saykin, Indiana Alzheimer's Disease Research Center, Indianapolis, IN; Indiana University School of Medicine, Department of Neurology, Indianapolis, IN
- Claudia Schulte, Hertie Institute for Clinical Brain Research, Neurodegenerative Diseases, Tübingen; German Center of Neurodegenerative Diseases , Department of Neurodegenerative Diseases, Tübingen
- Weiwei Schultz, Johnson & Johnson, JRD DSDH, Titusville, USA
- Geidy E Serrano, Banner Sun Health Research Institute, Sun City, Arizona, USA
- Nicholas T. Seyfried, Emory University School of Medicine, Atlanta, GA, USA; Emory University School of Medicine, Department of Neurology, Atlanta, GA, USA; Emory University School of Medicine, Department of Biochemistry, Atlanta, GA, USA
- Todd Sherer, Michael J. Fox Foundation, New York, NY, USA
- Bart Smets, Johnson & Johnson, Beerse, Belgium
- Emily Smith, Indiana Alzheimer's Disease Research Center, Indianapolis, IN; Indiana University School of Medicine, Department of Radiology & Imaging Sciences, Indianapolis, IN
- Adam M. Staffaroni, University of California, San Francisco, Neurology Department, San Francisco, CA
- Marc Suárez-Calvet, Barcelonaβeta Brain Research Center (BBRC), Pasqual Maragall Foundation, Barcelona, Spain; Hospital del Mar Research Institute, Barcelona, Spain; Hospital del Mar, Neurology Department, Barcelona, Spain
- Shinya Tasaki, Rush Alzheimer's Disease Center, Department of Neurological Sciences, Chicago, IL, USA
- Charlotte Teunissen, Neurochemistry Laboratory, Neurology Department, Amsterdam, the Netherlands; Amsterdam Neuroscience, Amsterdam, the Netherlands
- Qu Tian, NIH/NIA
- Betty M Tijms, Alzheimer Center Amsterdam, Neurology Department, Amsterdam, the Netherlands; Amsterdam Neuroscience, Amsterdam, the Netherlands
- Maarten Timmers, Johnson & Johnson, Beerse, Belgium

- Jigyasha Timsina, Washington University School of Medicine, Department of Psychiatry, St. Louis, 63110, MO, USA.; NeuroGenomics and Informatics Center, Washington University School of Medicine, St. Louis, 63110, MO, USA.
- Abolfazl Doostparast torshizi, Johnson & Johnson, Spring House, USA
- Sergi Valero, Ace Alzheimer Center Barcelona, Universitat Internacional de Catalunya, 08029 Barcelona, Spain; Biomedical Research Networking Centre in Neurodegenerative Diseases (CIBERNED), National Institute of Health Carlos III, 28029 Madrid, Spain
- Wiesje M van der Flier, Alzheimer Center Amsterdam, Neurology Department, Amsterdam, the Netherlands; Amsterdam Neuroscience, Amsterdam, the Netherlands; Epidemiology and Data Science, Amsterdam UMC
- Fernando Vieira, ALS Therapy Development Institute, Cambridge, MA, United States
- Natalia Vilor-Tejedor, Barcelonaβeta Brain Research Center (BBRC), Pasqual Maragall Foundation, Barcelona, Spain; Radboud University Medical Center, Department of Human Genetics, Nijmegen, Netherlands; Centre for Genomic Regulation (CRG), Barcelona Institute of Science and Technology (BIST), Barcelona, Spain
- Pieter Jelle Visser, Alzheimer Center Amsterdam, Neurology Department, Amsterdam, the Netherlands; Amsterdam Neuroscience, Amsterdam, the Netherlands; Alzheimer center Limburg, School for Mental Health and Neuroscience, Maastricht University
- Jacob Vogel, Department of Clinical Sciences Malmö, SciLifeLab, Lund University, Lund, Sweden
- Julia D. Webb, University of California, San Francisco, Neurology Department, San Francisco, CA
- Bryan K Woodruff, Mayo Clinic Arizona, Scottsdale, Arizona, USA
- Tony Wyss-Coray, Stanford University, The Phil and Penny Knight Initiative for Brain Resilience, Stanford, CA, USA; Stanford University, Wu Tsai Neurosciences Institute, Stanford, CA, USA; Stanford University School of Medicine, Department of Neurology and Neurological Sciences, Stanford, CA, USA
- Ying Xu, Washington University School of Medicine, Department of Psychiatry, St. Louis, 63110, MO, USA.; NeuroGenomics and Informatics Center, Washington University School of Medicine, St. Louis, 63110, MO, USA.
- Chengran Yang, Washington University School of Medicine, Department of Psychiatry, St. Louis, 63110, MO, USA.; NeuroGenomics and Informatics Center, Washington University School of Medicine, St. Louis, 63110, MO, USA.
- Mariet A. Younkin, Mayo Clinic, Neurology, Rochester, MN
