# Supplementary material for: APOE ε4 carriers share immune-related proteomic changes across neurodegenerative diseases
Source: Nat Med. 2025 Jul 15;31(8):2590–601. doi: 10.1038/s41591-025-03835-z (PMC12353839; doi:10.1038/s41591-025-03835-z)
Supplement: Supplementary file 2 — Reporting Summary [file 41591_2025_3835_MOESM2_ESM.pdf]

Corresponding author(s): Dr. Caitlin Finney & Dr. Artur Shvetcov

Last updated by author(s): Jun 5, 2025

Reporting Summary

Nature Portfolio wishes to improve the reproducibility of the work that we publish. This form provides structure for consistency and transparency in reporting. For further information on Nature Portfolio policies, see our Editorial Policies and the Editorial Policy Checklist.

Statistics

For all statistical analyses, confirm that the following items are present in the figure legend, table legend, main text, or Methods section.

n/a	Confirmed	
☐	☒	The exact sample size (*n*) for each experimental group/condition, given as a discrete number and unit of measurement
☐	☒	A statement on whether measurements were taken from distinct samples or whether the same sample was measured repeatedly
☐	☒	The statistical test(s) used AND whether they are one- or two-sided<br>*Only common tests should be described solely by name; describe more complex techniques in the Methods section.*
☐	☒	A description of all covariates tested
☐	☒	A description of any assumptions or corrections, such as tests of normality and adjustment for multiple comparisons
☐	☒	A full description of the statistical parameters including central tendency (e.g. means) or other basic estimates (e.g. regression coefficient) AND variation (e.g. standard deviation) or associated estimates of uncertainty (e.g. confidence intervals)
☐	☒	For null hypothesis testing, the test statistic (e.g. F, t, r) with confidence intervals, effect sizes, degrees of freedom and P value noted<br>*Give P values as exact values whenever suitable.*
☒	☐	For Bayesian analysis, information on the choice of priors and Markov chain Monte Carlo settings
☐	☒	For hierarchical and complex designs, identification of the appropriate level for tests and full reporting of outcomes
☒	☐	Estimates of effect sizes (e.g. Cohen's *d*, Pearson's *r*), indicating how they were calculated

Our web collection on statistics for biologists contains articles on many of the points above.

Software and code

Policy information about availability of computer code

Data collection	Data was collected from the Global Neurodegeneration Proteomics Consortium (https://www.neuroproteome.org/) v1 harmonized dataset.<br><br>Data was collected from the AMP-AD UPenn Proteomics Study data (https://adknowledgeportal.synapse.org/).
Data analysis	All code used for analyzing the proteomic and clinical data are available at https://github.com/Art83/gnpc_apoe.

For manuscripts utilizing custom algorithms or software that are central to the research but not yet described in published literature, software must be made available to editors and reviewers. We strongly encourage code deposition in a community repository (e.g. GitHub). See the Nature Portfolio guidelines for submitting code & software for further information.

Data

Policy information about availability of data

All manuscripts must include a data availability statement. This statement should provide the following information, where applicable:

- Accession codes, unique identifiers, or web links for publicly available datasets
- A description of any restrictions on data availability
- For clinical datasets or third party data, please ensure that the statement adheres to our policy

The harmonized GNPC data used to generate these findings was provided to Consortium Members in June 2024 and will be made available for public request by the AD Data Initiative by July 1, 2025. Members of the global research community will be able to access the metadata and place a data use request via the AD Discovery

Portal (https://discover.alzheimersdata.org/). Access is contingent on adherence to the GNPC Data Use Agreement and the Publication Policies.

The AMP-AD UPenn Proteomics Study data is available through the AD Knowledge Portal (https://adknowledgeportal.synapse.org/). Researchers who wish to access this controlled dataset are required to submit a Data Use Agreement. More information can be found here: https://adknowledgeportal.synapse.org/Data%20Access.

Research involving human participants, their data, or biological material

Policy information about studies with human participants or human data. See also policy information about sex, gender (identity/presentation), and sexual orientation and race, ethnicity and racism.

Reporting on sex and gender	Supplementary Tables 1 and 6 list the sex distributions for each group of individuals included in the study. We have also included an additional machine learning analysis to examine whether any sex-specific effects exist in our findings and show that there are none. Full machine learning performance metrics for this analysis are included in Extended Data Tables 1 and 2.
Reporting on race, ethnicity, or other socially relevant groupings	Supplementary Tables 1 and 6 list the race distributions for each group of individuals included in the study. We have also included an additional machine learning analysis to examine whether any race-specific effects exist in our findings and show that there are none (see Extended Data Table 2). We were limited by the races represented in the Global Neurodegeneration Proteomics Consortium dataset to White, Black or African American, and American Indian or Alaskan Native. Although additional races were represented, including Native Hawaiian or Other Pacific Islander and Asian, the N's were insufficient for a separate analysis.
Population characteristics	Supplementary Table lists the demographic and characteristic information available to us through the Global Neurodegeneration Proteomics Consortium dataset. Supplementary Table 6 lists the demographic and characteristic information available to us through the Accelerating Medicines Partnership in Alzheimer's Disease (AMP-AD) UPenn Proteomics Study cohort.
Recruitment	This study used data generated through the Global Neurodegeneration Proteomics Consortium which represents individuals recruited from 23 different clinical sites globally. The AMP-AD UPenn Proteomics study represents individual donors from the University of Pennsylvania School of Medicine Brain Bank.
Ethics oversight	Ethics approval was obtained independently by each of the sites contributing data to the Global Neurodegeneration Proteomics Consortium dataset and the AMP-AD UPenn Proteomics Study dataset. Details on contributing sites can be found in Imam et al. (2025) Nature Medicine

Note that full information on the approval of the study protocol must also be provided in the manuscript.

Field-specific reporting

Please select the one below that is the best fit for your research. If you are not sure, read the appropriate sections before making your selection.

☒ Life sciences ☐ Behavioural & social sciences ☐ Ecological, evolutionary & environmental sciences

For a reference copy of the document with all sections, see nature.com/documents/nr-reporting-summary-flat.pdf

Life sciences study design

All studies must disclose on these points even when the disclosure is negative.

Sample size	We did not include a sample size calculation as we were limited by the number of individuals represented in the Global Neurodegeneration Proteomics Consortium dataset and the AMP-AD UPenn Proteomics datasets. We included all individuals possible who had full genotyping data for APOE variants. This resulted in a final N of 11,270 from the Global Neurodegeneration Proteomics Consortium and 262 from the AMP-AD UPenn Proteomics Study. This was the maximum N available to us.
Data exclusions	We included all individuals from the Global Neurodegeneration Proteomics Consortium and AMP-AD UPenn Proteomics datasets who had full APOE genotyping available. This was the only exclusion criteria and we did not perform any post-hoc exclusions in our study.
Replication	In our study, we used machine learning to replicate the APOE4 proteome signatures found across the different neurodegenerative disease groups and report full metrics of this in Extended Data Tables 1 and 2. We also use enrichment analyses to determine the replication of represented biological pathways and functions across the cerebrospinal fluid, plasma, and brain and include FDR statistics for this in Supplementary Tables 3, 5, and 8. Further, we replicate our finding from the cerebrospinal fluid and plasma from the Global Neurodegeneration Proteomics Consortium in the brains from a different cohort of individuals from the AMP-AD UPenn Proteomics Study cohort. We also provide cross-platform, orthogonal validation of the SomaScan assay enriched pathways through label free mass spectrometry-identified enriched pathways.
Randomization	Participants were allocated to groups based on the clinical diagnosis of different neurodegenerative diseases including AD, FTD, PDD, PD, ALS, and non-impaired controls. To facilitate machine learning model training and evaluation individuals were randomly divided into training (70%) and withheld testing (30%) datasets using a pre-defined random seed ensuring the results are reproducible.
Blinding	The investigators were not blind to the individual diagnostic groups. Blinding was not relevant to our study because we use machine learning models which are inherently blinded to the group allocation in the testing dataset. Only blinded testing metrics are reported in our study.

Reporting for specific materials, systems and methods

We require information from authors about some types of materials, experimental systems and methods used in many studies. Here, indicate whether each material, system or method listed is relevant to your study. If you are not sure if a list item applies to your research, read the appropriate section before selecting a response.

Materials & experimental systems

n/a	Involved in the study
☒	☐ Antibodies
☒	☐ Eukaryotic cell lines
☒	☐ Palaeontology and archaeology
☒	☐ Animals and other organisms
☒	☐ Clinical data
☒	☐ Dual use research of concern
☒	☐ Plants

Methods

n/a	Involved in the study
☒	☐ ChIP-seq
☒	☐ Flow cytometry
☒	☐ MRI-based neuroimaging

Plants

Seed stocks	*Report on the source of all seed stocks or other plant material used. If applicable, state the seed stock centre and catalogue number. If plant specimens were collected from the field, describe the collection location, date and sampling procedures.*
Novel plant genotypes	*Describe the methods by which all novel plant genotypes were produced. This includes those generated by transgenic approaches, gene editing, chemical/radiation-based mutagenesis and hybridization. For transgenic lines, describe the transformation method, the number of independent lines analyzed and the generation upon which experiments were performed. For gene-edited lines, describe the editor used, the endogenous sequence targeted for editing, the targeting guide RNA sequence (if applicable) and how the editor was applied.*
Authentication	*Describe any authentication procedures for each seed stock used or novel genotype generated. Describe any experiments used to assess the effect of a mutation and, where applicable, how potential secondary effects (e.g. second site T-DNA insertions, mosiacism, off-target gene editing) were examined.*
